# Terminology, Taxonomy, and Facilitation of Motor Learning in Clinical Practice: Protocol of a Delphi Study

**DOI:** 10.2196/resprot.2604

**Published:** 2013-05-17

**Authors:** Melanie Kleynen, Michel HC Bleijlevens, Anna JHM Beurskens, Sascha M Rasquin, Jos Halfens, Mark R Wilson, Rich S Masters, Monique A Lexis, Susy M Braun

**Affiliations:** ^1^Research Centre Autonomy and Participation of People with a Chronic IllnessFaculty of HealthZuyd University of Applied SciencesHeerlenNetherlands; ^2^AdelanteCentre of Expertise in RehabilitationHoensbroekNetherlands; ^3^Department of Health Services Researchschool CAPHRIMaastricht UniversityMaastrichtNetherlands; ^4^Centre of Expertise Geriatric Rehabilitation and Chronic Somatic CareSevagram ZorgcentraHeerlenNetherlands; ^5^Department of General PracticeFaculty of Health, Medicine and Life SciencesMaastricht UniversityMaastrichtNetherlands; ^6^Department of Rehabilitationschool CAPHRIMaastricht UniversityMaastrichtNetherlands; ^7^Department of Sport and Health SciencesUniversity of ExeterExeterUnited Kingdom; ^8^Institute of Human PerformanceUniversity of Hong KongHong Kong SARChina; ^9^Research Centre Technology in CareFaculty of HealthZuyd University of Applied SciencesHeerlenNetherlands

**Keywords:** motor learning, Delphi technique, clinical practice, consensus, definitions

## Abstract

**Background:**

Facilitating motor learning in patients during clinical practice is complex, especially in people with cognitive impairments. General principles of motor learning are available for therapists to use in their practice. However, the translation of evidence from the different fields of motor learning for use in clinical practice is problematic due to lack of uniformity in definition and taxonomy of terms related to motor learning.

**Objective:**

The objective of this paper was to describe the design of a Delphi technique to reach consensus on definitions, descriptions, and taxonomy used within motor learning and to explore experts’ opinions and experiences on the application of motor learning in practice.

**Methods:**

A heterogeneous sample of at least 30 international experts on motor learning will be recruited. Their opinions regarding several central topics on motor learning using a Delphi technique will be collected in 3 sequential rounds. The questionnaires in the 3 rounds will be developed based on the literature and answers of experts from earlier rounds. Consensus will be reached when at least 70% of the experts agree on a certain topic. Free text comments and answers from open questions on opinions and experiences will be described and clustered into themes.

**Results:**

This study is currently ongoing. It is financially supported by Stichting Alliantie Innovatie (Innovation Alliance Foundation), RAAK-international (Registration number: 2011-3-33int).

**Conclusions:**

The results of this study will enable us to summarize and categorize expert knowledge and experiences in a format that should be more accessible for therapists to use in support of their clinical practice. Unresolved aspects will direct future research.

## Introduction

### Background

Motor learning has been a central topic in the sport domain, and has more recently received increased attention in the context of rehabilitation [1], especially in people with neurological disorders [2,3]. In both populations, research into fundamental (eg, underlying mechanisms) [4] as well as clinical (eg, application to individuals) aspects [5,6] of motor learning is increasing. Although the target populations within sport and rehabilitation do not seem to be comparable, the processes, principles, and underlying assumptions of their learning process share considerable features. However, a clear structure for the translation of knowledge and evidence, not only from sports to rehabilitation, but also from laboratory research to the clinical situation, is currently absent.

### Speaking the Same Language

Within the behavioral motor learning literature, usually in the context of skill acquisition in sports, several models and concepts exist where different terms, classifications, and/or taxonomies are used (eg, [7-13]). Often, the degree to which conscious knowledge is involved in the learning process is used as a starting point. Forms of learning that result in the accumulation of non-conscious, procedural knowledge are described as implicit, whereas forms of learning that result in the accumulation of conscious, declarative knowledge are generally described as explicit [14,15]. In recent years, there has been a significant increase in the number of studies evaluating the application of implicit and explicit forms of learning. Target populations are not only healthy people and athletes but also patients with neurological disorders [16-26].

Unfortunately, there is a lack of clarity with regard to definitions across studies and consequently the forms of learning are applied differently within study paradigms.

If we want to link research from different fields, we need to enable comparison of evidence and expertise. In order to further translate results into practice, it is important that researchers, therapists, and others professionals involved in facilitating the motor learning process speak the same language and use uniform terminology. Therefore, the main aim of the described study protocol is to achieve consensus on the definitions, descriptions, and taxonomy of terms related to motor learning, using the distinction in implicit and explicit forms of motor learning as a conceptual basis.

### Application of Motor Learning

Physiotherapists and occupational therapists are specialized in providing therapy that is tailored to facilitate motor skill learning of patients with a wide range of pathologies. A substantial proportion of the patients therapists treat are older people with pathologies of the central nervous system, related to conditions such as stroke, Parkinson’s disease or dementia [27]. As well as motor problems, these patients often experience problems on a cognitive level, making motor learning more difficult [28].

Some general principles of motor learning related to neural plasticity (eg, intensive and task specific training, “use it or lose it”) are available for therapists to use in their practice [29,30]. These principles generally direct clinical practice in terms of what to do and how often; however, the application of these theoretical principles during daily practice often remains unclear (eg, When and how to vary between tasks? Which instructions should be given and when?).

Traditionally, therapists often use rational arguments and many verbal instructions to engage patients in motor learning [31] possibly promoting more explicit forms of motor learning. In patients with cognitive impairments, this approach is often not feasible. It remains unclear though to what extent cognitive impairments should influence the choice between more implicit and more explicit forms of learning [32].

Achieving consensus on applying motor learning is probably not realistic and maybe even not desirable, as clinical practice is complex and choices made within the motor learning process are often multi-factorial. Following a “one-size-fits-all” approach to motor learning is not possible in such a dynamic process. However, especially for less experienced therapists, it is important to have a starting point, a framework, which can help guide their practice while leaving enough space for patient tailored decision-making. The second aim of the study is therefore to explore how motor learning can be facilitated in practice and how choices for motor learning strategies can be made, particularly in people with cognitive impairments. The experiences of the experts might provide indications of how theory can be translated into practice and provide a framework to support therapists’ choices for designing treatment.

The objective of this paper was to describe the design of a Delphi technique: (1) to achieve consensus on the definitions, descriptions, and taxonomy of terms related to motor learning, and (2) to explore how motor learning can be facilitated in practice and how choices within motor learning can be made, using the distinction in implicit and explicit forms of motor learning as a conceptual basis.

## Method

### Delphi Technique

The Delphi technique consists of a series of sequential questionnaires or “rounds” aiming to obtain the most reliable consensus of opinions from a group of experts [33]. The Delphi technique was chosen because it is useful for situations where individual opinions and knowledge are selected, compared, and combined in order to address a lack of agreement or an incomplete state of knowledge [33,34]. In this study, at least 30 experts will be invited to provide their opinion of different motor learning-related constructs. Two parallel processes will be initiated in the preparation of the actual Delphi rounds: (1) identification and invitation of experts, and (2) design of the structure and content of the questionnaires in the Delphi rounds.

### Referee Group

An international referee group, consisting of all authors of this paper, will identify and invite the experts. We will also prepare the content of the Delphi rounds and will supervise and monitor the process. We are a group of 7 researchers and 2 therapists with expertise in the field of motor learning and/or conducting the Delphi technique. Our backgrounds include epidemiology, physiotherapy, occupational therapy, movement sciences, and (sport) psychology. As members of the referee group, we will not participate in the survey.

### Identification and Invitation of Experts

**Figure 1:**
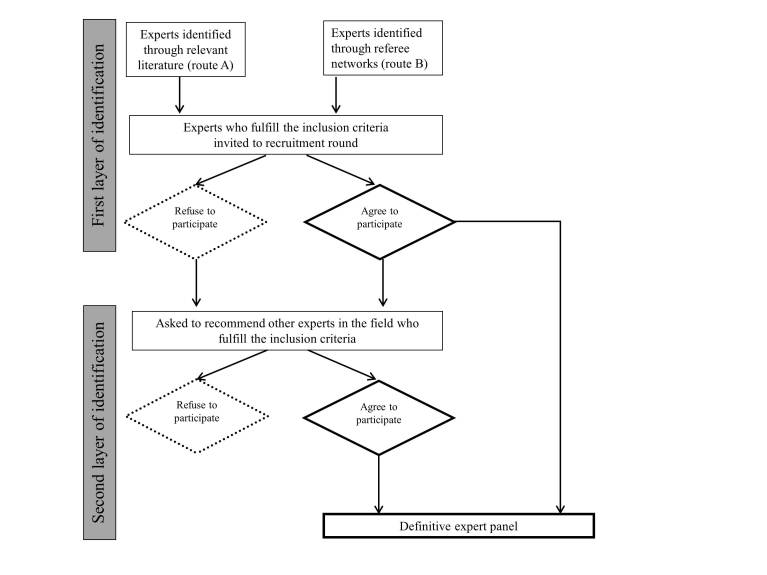
Identification and composition of the expert panel.

Heterogeneity within the expert panel is an important quality criterion [33]. We will therefore seek to include experts from different fields of motor learning. These experts should be researchers, lecturers, experienced therapists, or coaches working in the field of motor learning. Figure 1 provides an overview of how the experts will be identified and the expert panel will be composed. Experts in the field of research will be identified through a literature search (Figure 1, route A). The referee group will identify lecturers, experienced therapists, and coaches using their networks as these experts are more difficult to identify through literature (Figure 1, route B). Both routes together will be termed the *first layer of identification*. The aim of the extensive selection procedure is to create a heterogenic, international expert panel. However, it is not possible to predict to what extent we will succeed, as the expert group will be a purposive sample and not stratified on all characteristics that might be of influence.

#### Experts Identified From Literature (Route A)

Researchers in the field of motor learning will be identified by an extensive literature search. This search will be conducted through PubMed/Medline and PsycINFO. Several search terms will be combined, depending on the search options of the digital database. The most important search terms will be motor learning, implicit, explicit, and skill acquisition. A researcher will be defined as an expert if he/she is the first, second, or last author of at least one empirical publication in the area of motor learning. Publications can be in the field of motor learning or skill acquisition in healthy populations, sports, and rehabilitation. Experts who have only published in the field of fundamental neuroscience related to motor learning will not be invited to participate, as the focus of the Delphi study is on facilitating motor learning in clinical practice. Fundamental research will be defined as studies using only outcome measures evaluating “body function and structures”, according to the International Classification of Functioning, Disability, and Health [35].

#### Experts Identified From the Referee Members’ Network (Route B)

Parallel to the identification through the literature search, experts with practical expertise, such as therapists, lecturers, and coaches, will be recruited from the networks of the referee group. Though somewhat arbitrary, we defined an expert as a therapists, coach, or lecturer with at least 3 years of working experience in applying motor learning in practice and involvement in education or research.

### Recruitment Round

All eligible experts will be invited to participate in a recruitment round. Experts will receive an email comprising of a brief introduction of the aim and content of the survey, the amount of time to complete the questionnaires, and a personal link to open the online survey program. The aim of this recruitment round will be twofold. The first aim is to inform experts about the survey and to obtain consent for participation. Participating experts will be asked to provide detailed information on their age, background, years of experience, field of interest, working country, and current position to help to define the composition of the panel (see Multimedia Appendix 1). The second aim is to identify additional experts who were not identified through the literature and the network of the referee group members. All invited experts will be asked to recommend other experts (Figure 1, the so-called *second layer of identification*) irrespective of whether they have agreed to participate or not (ie, snow-ball sampling). They will be explicitly asked to identify expert lecturers, coaches, or therapists who fulfill the inclusion criteria, as those experts are more difficult to identify through publications. This process hopes to limit the extent to which the sample of experts is biased by the network of the referee group.

### Panel Size and Composition

There are no clear guidelines for an appropriate panel size for studies using the Delphi technique and there is only limited evidence on the effect of the panel size on the validity and reliability of any consensus that is reached [34]. Therefore, in accordance with another study [36], we consider a panel size of at least 30 experts to be appropriate—approximately 10 researchers from motor learning in rehabilitation, 10 researchers from the field of motor learning in healthy individuals and sports, and 10 experts with experience in applying motor learning in practice. Although it is not possible to predict the number of experts who will be identified, agree to participate, and complete the survey, we used data from earlier studies for guidance. Based on data of a recent, Web-based Delphi study [37], it is expected that 60% of the invited experts will agree to participate, that 70% of the participants will return the first questionnaire, and 50% of the participants will complete the entire survey. Therefore, we will initially invite at least 100 experts to participate (on a voluntary basis), however no upper limit will be imposed on the number of invited experts. Experts who do not respond to the invitation will be reminded twice to do so. If experts agree to participate, they will be considered part of the definitive expert panel. Experts who agree to participate but do not respond to one of the questionnaires will be sent two reminders. As long as experts do not explicitly withdraw from participation (via mail or using a link within the survey), they will be considered part of the panel and will receive an invitation for each round. An exception will be those experts who do not respond to round one and round two. They will not be invited to the third round and will be excluded from the panel.

### Design and Content of the Survey

**Figure 2:**
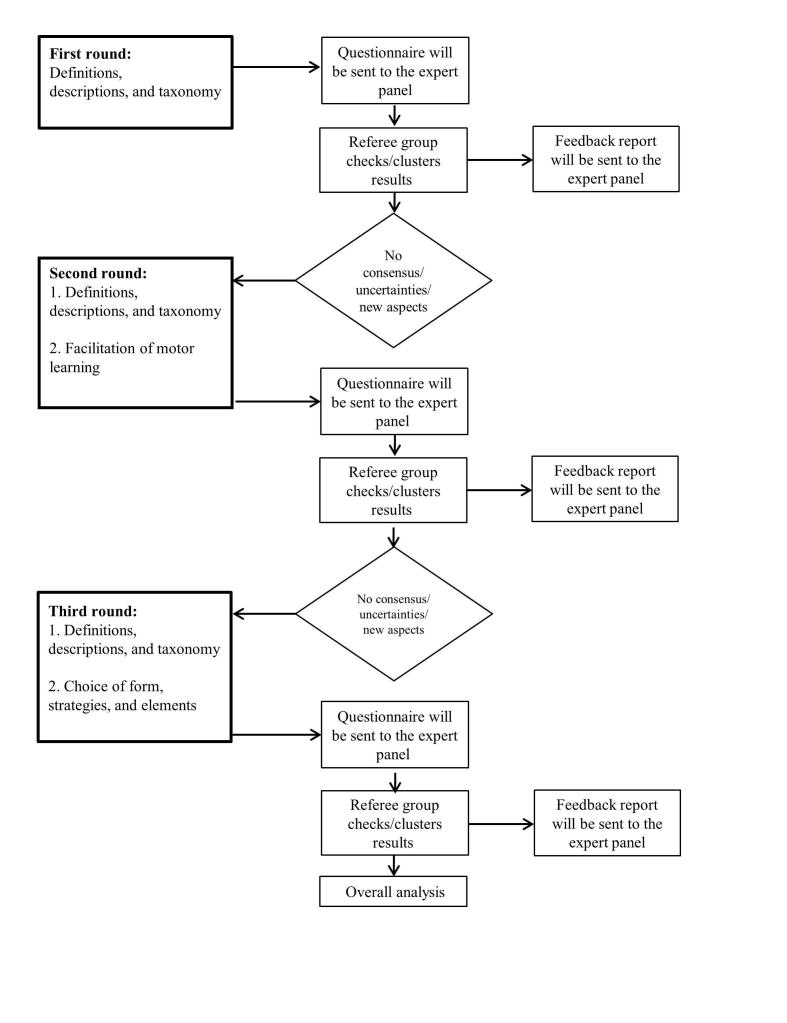
Overview of the procedure and content of the Delphi rounds (squares=process steps; rhomboids=decision steps).

All rounds will be designed and distributed using an online survey program (SurveyMonkey, LLC, California, USA). Figure 2 provides an overview of the process and content of the 3 rounds. In the following section, the content of the 3 rounds and the expected results is described. The description of the first round is more detailed than the second and third rounds, as the content of these rounds will mainly be based on the findings from the earlier ones. In general, the second and third round will each consist of 2 parts. In the first part, answers from the former round will be further verified and the second part will focus on new aspects.

### The First Round

The first round will focus on the definitions, descriptions, and taxonomy of implicit and explicit forms of motor learning and a variety of motor learning strategies.

First, aspects of different definitions and descriptions for implicit and explicit motor learning that are provided in the literature will be presented. Experts will be asked to choose which of these aspects should be included in the definitions. Next, a list of strategies (eg, analogy learning, discovery learning) that are often described in the literature will be presented together with a description of each strategy. Per strategy, experts will first be asked whether they know the strategy and whether they have used the strategy in research or in practice. Experts, who stated to know the strategy, will then be asked whether they agree with the description provided. If they do not agree, they will be asked to provide arguments in an open comment box. Third, experts will be asked whether they can classify the strategy as promoting a more implicit or explicit form of motor learning.

### Preliminary Data Analysis After First Round

To prepare the second round, the referee group will perform a preliminary analysis of data. Definitions of implicit and explicit motor learning will be created based on consensus from the separate definitional aspects provided in the survey. Consensus will be defined when 70% or more of the experts agree on a certain aspect. If no consensus is achieved, then percentages of agreement will be presented, however, no definitions will be formulated. Only strategies that more than 70% of the experts state to know will be taken into account in the second round (termed best-known strategies). Descriptions of those strategies will be adapted and if necessary, reformulated based on the open text comments.

### The Second Round

The aim of the second round will be twofold. First, a summary of the answers of the first round will be provided. The formulated definitions will be presented to the experts and they will be asked whether they agree with these definitions. The adapted description of the strategies will also be presented again.

The second part of the survey will focus on experts’ opinions and experiences on how motor learning can be facilitated in a single therapy session. Experts will be asked to state how instructions, feedback, and organization of the environment (so-called “elements” of motor learning) can be used to facilitate implicit and explicit learning.

Experts will be presented with a list including elements that could be used to facilitate motor learning. They will be asked whether these elements would facilitate a more implicit or a more explicit form of learning. To make answers comparable, we will mainly use multiple choice questions, however, experts will have the opportunity to comment on every question (either by using the option “other” or “open comment box”). Furthermore, we will assess how these elements relate to the motor learning strategies identified by the experts as the best-known strategies from round one.

### The Third Round

If necessary, aspects for which no consensus in definitions, descriptions, and taxonomy was reached in rounds one and two will be presented again. Further, the second aim of the third round will be the identification of factors influencing and directing choices made within the motor learning process. The impact of cognitive impairments for these choices will be addressed specifically.

### Data Analysis

The referee group will be unaware of the identity of expert panel members with the exception of two members of the referee group who are responsible for correspondence (MK, SB). The analysis of the responses of the experts will be processed anonymously.

The questionnaires for the 3 rounds will consist of closed/multiple choice questions and some open questions. Closed/multiple choice questions will be used if there is some knowledge available with regard to the answers (eg, from the literature or earlier survey rounds). Each closed/multiple choice question will have the option “other” or “comment” to ensure that experts can also add answers that are not listed. If little or not enough knowledge is available to pre-structure the answer options, open questions will be used. Further, open questions will be used to inventory experiences of the expert panel.

The referee group will not decide for specific aspects where no consensus is reached. They will however, choose between two different options to proceed: (1) the aspect will be presented again to the expert panel in cases where consensus is likely to be achieved in the next survey round, or (2) the variety in answers will be reported in case of very diverse answers.

The answer to all explorative questions (facilitation of motor learning in round 2, and choice of form, strategies, and elements in round 3) will be analyzed using majorities and trends (eg, ≥ 50%). Consensus is not expected for these questions as answers will be more influenced by the specific practical experience the expert has, and the target group he/she works with. Free text comments and answers from open questions will be described and if possible, clustered into themes. Quotes will be used to illustrate the main results.

### Feedback Reports

After every round, a summary of the results will be sent to each member of the expert panel. The results will be clustered, but not analyzed or interpreted in detail.

## Results

This study is currently ongoing. It is financially supported by Stichting Alliantie Innovatie (Innovation Alliance Foundation), RAAK-international (Registration number: 2011-3-33int).

## Discussion

This paper describes the design of a study using the Delphi technique in the broad area of motor learning. To our knowledge, it is the first time that the Delphi technique has been used for this topic area. The objective of this paper was to describe the design of the Delphi technique to reach consensus on definitions, descriptions, and taxonomy used within motor learning and to explore experts’ opinions and experiences on the application of motor learning in practice. However, as in any other study designs, the Delphi technique is subject to some points of consideration.

The most important advantage of using the Delphi technique is that it enables the synthesis of existing knowledge from experts with different backgrounds, including unpublished and practical expertise. In addition to gaining more insight into the definitions and taxonomy used within motor learning, the results of this study might also shed light on unresolved questions and controversial aspects within the field. A disadvantage of the Delphi technique is that the questions and answers are generally based on a theoretical, hypothetical basis. In addition, the referee group needs to have some conceptual structure in designing the survey. In this study, the distinction in implicit and explicit forms of motor learning is used, which will probably influence the line of reasoning and answers of the participants to some extent.

A well-composited expert panel is the linchpin of this study. As the scope of the Delphi topic is broad, it is important that the expert panel truly represents the available expertise on the subject. Experts from different fields of motor learning and with different backgrounds must participate in the Delphi study. As invited experts will be asked to recommend other experts, we will try to invite as broad a sample of experts as possible to prevent selection bias, however, only after the results are available can a judgment of the representativeness of the expert panel be made.

No new evidence will be generated by this study. The Delphi technique will merely be used to summarize existing knowledge and experiences regarding motor learning from experts with different backgrounds. It is therefore important that the results of this study will be considered as a starting point for future applied research. The aim of this research should be to confirm results and further explore unresolved aspects found in this study. At the same time, the available knowledge and experiences from the experts in this study can be accessed by therapists (and other users) who might find the information useful to directly support their clinical reasoning and practice.

## Multimedia Appendix 1

Example from the online survey.
