# Supplementary figures and images for: Terminology, Taxonomy, and Facilitation of Motor Learning in Clinical Practice: Protocol of a Delphi Study
Source: JMIR Res Protoc. 2013 May 17;2(1):e18. doi: 10.2196/resprot.2604 (PMC3668605; doi:10.2196/resprot.2604)

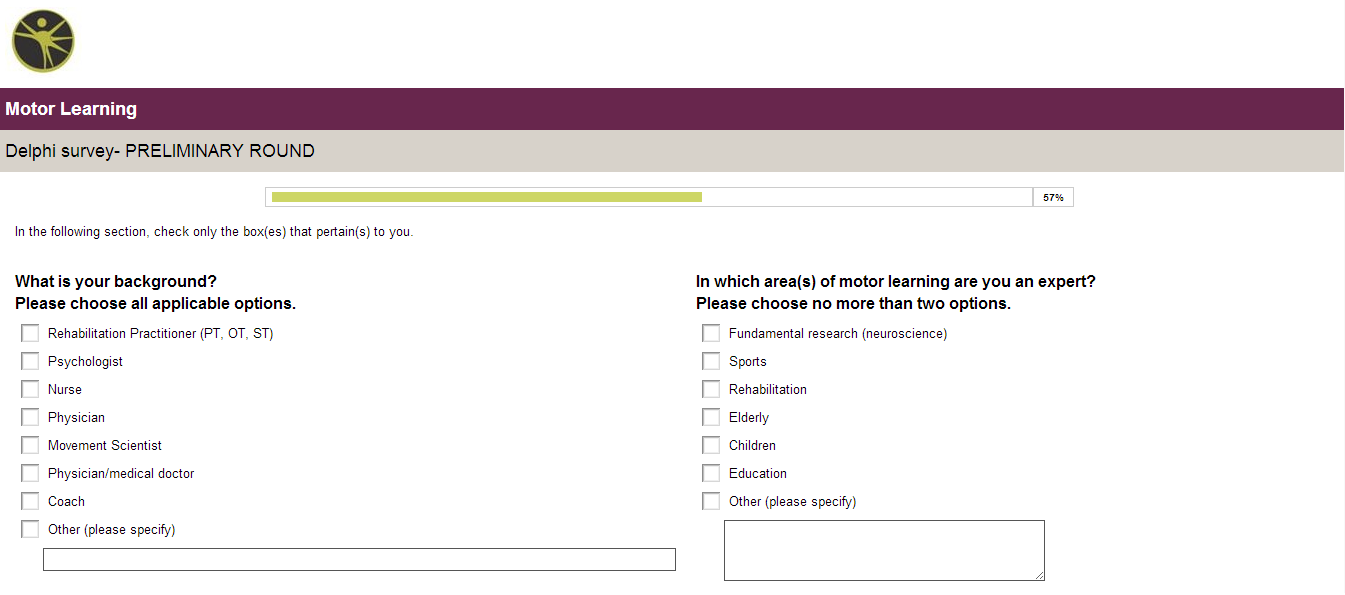

Supplement: Supplementary file 1 [file resprot_v2i1e18_app1.png]
